# Mothers and fathers who use medical cannabis: A comparison of sociodemographic characteristics, cannabis use, and health

**DOI:** 10.1016/j.dadr.2025.100390

**Published:** 2025-10-24

**Authors:** Janna Ataiants, Ekaterina V. Fedorova, Ojaswini D. Parab, Maddy Finkelstein, Elizabeth S. Valdez, Olivia Cordingley, Stephen E. Lankenau

**Affiliations:** Drexel University, Dornsife School of Public Health, Department of Community Health and Prevention, 3215 Market Street, Philadelphia, PA 19104, United States

**Keywords:** Mothers and fathers, Gender differences, Medical cannabis patients, Medical cannabis use

## Abstract

**Background:**

While increasing numbers of parents use medical cannabis, little is known about their cannabis use or health, despite potential implications for child well-being. Recognizing that parenting is shaped by gendered roles and norms, this study compared mothers and fathers who use cannabis as medical cannabis patients.

**Methods:**

Parents living with children (N = 372; 62.9 % mothers, 37.1 % fathers) were identified from an ongoing study of Pennsylvania-based medical cannabis patients. Bivariate and multivariable analyses assessed sociodemographic, cannabis use, and health characteristics by parent’s sex.

**Results:**

Compared to fathers, mothers were less likely to be married, have a higher income, report arrest history, veteran status, and lifetime opioid misuse, and more likely to report PTSD (all *p* < 0.05). Problematic cannabis use did not significantly differ by parent’s sex (mothers: 15.0 %; fathers: 20.3 %). However, mothers had lower odds of using concentrates (AOR: 0.56, 95 % CI: 0.33–0.96) and higher odds of using capsules (AOR: 1.87, 95 % CI: 1.09–3.19), tinctures (AOR: 1.71, 95 % CI: 1.01–2.87), using at specific times (AOR: 1.85, 95 % CI: 1.08–3.16), and using alone (AOR: 1.79, 95 % CI: 1.02–3.14). Mothers also had higher odds of frequent sleep problems (AOR: 1.96, 95 % CI: 1.18–3.25) and moderate-to-severe anxiety (AOR: 1.91, 95 % CI: 1.14–3.20).

**Discussion:**

Mothers showed greater socioeconomic vulnerability, elevated anxiety and sleep disturbances, and more discreet cannabis use, while fathers had more flexible use patterns and fewer anxiety symptoms, despite greater lifetime adversity. These differences highlight the need for gender-responsive programs to support parents who use medical cannabis.

## Highlights

• Mothers had higher socioeconomic vulnerability, anxiety, and sleep problems.
• Mothers more often used oral cannabis and used more discreetly.
• Fathers had greater lifetime adversity but reported lower anxiety.
• Fathers more often used concentrates and had more flexible use patterns.
• Overall, only 17 % had problematic use, with no sex differences among parents.

## 1 Introduction

With cannabis legalization expanding in the United States (Boehnke et al., 2024), a growing number of adults, including parents, are becoming medical cannabis patients. Although medical cannabis registries do not record parental status, national survey data indicate that in medical-only states, a notable proportion of adults living with children report past-month (9.3 %) and daily (3.2 %) cannabis use. These rates are lower than in states with adult-use cannabis (11.9 % and 4.2 %, respectively) but higher than in states where cannabis remains illegal (6.1 % and 2.3 %) (Goodwin et al., 2021). Notably, a recent study pointed to potential benefits of medical cannabis laws for parenting, such as increased active time parents spent with their children following legalization (Bansak and Kim, 2024). Despite these developments, the experiences of parents who use medical cannabis legally remain understudied, even though their well-being directly affects the health, safety, and daily lives of their children.

Prior research has primarily focused on various risks associated with parental cannabis use, including adverse perinatal outcomes (Baia and Domingues, 2024), accidental pediatric exposure (Dean et al., 2021), or parent-to-offspring transmission of use (Madras et al., 2019). Concerns have also been raised about potential links between parental use and child maltreatment (Freisthler et al., 2015), although a recent systematic review found no consistent evidence of such effects (Wilson and Rhee, 2022). However, much of this literature does not distinguish between legal medical and other forms of cannabis use. To date, only a few qualitative studies have examined parents’ experiences in legal medical contexts, reporting perceived improvements in both health and parenting (Finkelstein et al., 2025, Thurstone et al., 2013, Valdez et al., 2025, Withanarachchie et al., 2025). Yet, quantitative research describing health and legal medical cannabis use among parents remains notably lacking.

Gender likely shapes how parents experience medical cannabis use, as mothers and fathers navigate distinct caregiving roles, social expectations, and health-related challenges. In the general population, mothers continue to assume a larger share of domestic labor and child-rearing responsibilities (Dunatchik et al., 2021, Pew Research Center, 2021) and report greater work-family conflict (Young et al., 2023), higher levels of stress (Musick et al., 2016), a greater prevalence of mental illness (Stambaugh et al., 2017), and greater perceived social judgment regarding their parenting (Pew Research Center, 2023) than fathers. Among medical cannabis patients, both mothers and fathers experience stigma and scrutiny (Finkelstein et al., 2025, Malleis, 2011). Yet mothers may face greater consequences, as U.S. institutional policies tend to treat both prenatal and general maternal substance use as grounds for state intervention. Prenatal use is often singled out for especially punitive responses, such as mandatory reporting and, in some states, criminal prosecution (Austin et al., 2022, Mills, 2022, Paltrow and Flavin, 2013), while general maternal use is more often linked to increased child welfare involvement and potential custody loss (Saunt and Montoya, 2025). Some U.S. states with medical cannabis laws, including Pennsylvania, provide certified patients with limited legal protections, specifically, that authorized medical cannabis use cannot, by itself, be considered by a court in custody determinations (Commonwealth of Pennsylvania, 2016). However, mandatory reporting laws for prenatal cannabis use continue to be enforced even after legalization (Krening and Hanson, 2018). Evidence from other countries similarly indicates that mothers continue to experience stigma and fear of surveillance despite cannabis legalization (Greene et al., 2023, Withanarachchie et al., 2025).

Despite these gendered dynamics, research on parental cannabis use rarely disaggregates findings by sex or gender and often lacks contextual detail about how cannabis is used, specifically, whether it is used in safer or more harmful ways (Pedersen et al., 2016). This gap limits our understanding of whether and how gendered caregiving roles shape cannabis use practices, which may carry important implications for parenting and child well-being. One key concern is whether parent’s gender is associated with problematic or disordered cannabis use, since cannabis use disorder, rather than any use, has been associated with impaired parenting (Hill et al., 2018). Another important dimension involves the context and timing of use, particularly whether cannabis is used in the presence of children or during specific times of the day. While use in the presence of children is often assumed, qualitative studies suggest that parents often use discreetly (Finkelstein et al., 2025, Thurstone et al., 2013, Valdez et al., 2025) and a recent ecological momentary assessment found that 89 % of use events occurred away from children, including 46 % when parents were alone (Freisthler et al., 2024). Product type also matters, as cannabis concentrates, for example, due to their potency and rapid onset, are associated with elevated risk of cannabis use disorder (Inman and Cservenka, 2024). Broader research shows that men are more likely to engage in frequent use and to prefer combustible or potent products (e.g., concentrates, joints), while women tend to choose products that can be used more discreetly (e.g., edibles, pipes), reflecting differences in both experience and stigma (Cuttler et al., 2016). Whether these gendered patterns apply to parents remains unknown.

Health challenges may further shape the functioning and caregiving capacity of parents who use medical cannabis. By definition, all medical cannabis patients live with at least one chronic condition, and conditions such as chronic pain (Wilson and Fales, 2015) and mental illness (Smith, 2004) including PTSD (Stover, et al., 2012), have been shown to interfere with parenting. In the general U.S. population, three common qualifying conditions for medical cannabis are more prevalent among women than men, including chronic pain (Lucas and Sohi, 2024), PTSD (Olff, 2017), and anxiety disorders (McLean et al., 2011), though current data on anxiety disorders reflect earlier classification systems that included PTSD. While opioid use disorder is not widely approved as a qualifying condition, some jurisdictions, including Pennsylvania, recognize it, and historically, it has been more prevalent in men (McHugh et al., 2021). Still, it is unknown whether these gender differences persist among parents who use medical cannabis, and more importantly, how they translate into current health challenges, including anxiety, depression, and sleep problems, which are central to parenting.

This study addresses these gaps by examining whether and how mothers and fathers who use medical cannabis differ in their health conditions, cannabis use behaviors, and social backgrounds. This study focuses specifically on mothers and fathers who use medical cannabis, a subgroup with distinct public health relevance. Drawing on data from Pennsylvania medical cannabis patients who are parents, we explore how gender shapes the ways medical cannabis is experienced in the context of caregiving. By identifying gendered patterns in use and health among parents, we aim to better understand parental health needs, cannabis practices, and family environments that shape children’s daily lives.

## 2 Methods

### 2.1 Study

This analysis uses baseline data from an ongoing, rolling cohort study of medical cannabis patients in Pennsylvania, approved by Drexel University's Institutional Review Board. Between June 2021 and March 2025, 1142 participants were recruited from Zen Leaf dispensaries across the state. Eligible participants were 18 or older and enrolled in Pennsylvania's Medical Marijuana Program, which accepts people with at least one of 24 qualifying medical conditions certified by a physician (Commonwealth of Pennsylvania, 2025). All participants provided informed consent. Data collection involved a baseline survey and quarterly follow-up assessments, administered via REDCap.

For this cross-sectional analysis, we focused on active parents, defined as participants who lived with their children. Of the 1142 patients, 1137 reported parental status: 404 were parents living with children, 192 parents not living with children, and 541 were nonparents. Parents living with children were on average younger than parents not living with children but older than nonparents, and were more likely to be female and married (all *p* < .05, data not shown). Other demographic differences were not significant. After excluding 32 participants with missing data on key baseline variables, the final analytical sample included 372 active parents.

### 2.2 Measures

#### 2.2.1 Sociodemographic and baseline characteristics

Participants reported sociodemographic characteristics, including age, race, sex, relationship status, income, education, past 90-day employment, veteran status, and arrest history, including cannabis possession arrests. Although sex variable (female/male) was used to distinguish between mothers and fathers, our interpretation is informed by gendered roles and norms that shape parenting (Yaffe, 2023).

Race was categorized as White versus non-White due to the predominance of participants identifying as non-Hispanic White. Relationship status was coded as “married” versus “not married,” combining all non-marital categories due to lack of cohabitation data. Income was dichotomized as below or above $50,000 annually, and education as “less than a Bachelor’s degree” versus “Bachelor’s degree or higher,” which approximately split the sample evenly. Participants also reported on their substance use and health histories. Lifetime cannabis use was assessed with questions about age at onset, history of daily or regular use (yes/no), and whether cannabis use began before receiving a doctor’s certification for medical cannabis (i.e., whether participants were naïve to cannabis use). Lifetime opioid misuse was measured as a composite variable encompassing any nonmedical use of heroin, fentanyl, or prescription opioids, which were initially assessed as separate items. Participants also reported lifetime diagnoses of chronic conditions, including PTSD, neuropathic, chronic, or intractable pain, and drug or alcohol dependence (including opioid use disorder). In addition, participants were asked about any of 24 qualifying health conditions they disclosed to a certifying physician to obtain their current certification for medical cannabis in Pennsylvania.

#### 2.2.2 Past 90-day cannabis use

Cannabis use frequency over the past 90 days was assessed with a four-category measure: 5–7 days per week (i.e., daily/near-daily), 1–4 days per week (i.e., weekly), 1–3 days per month (i.e., monthly), and less than once per month. For regression analysis, the measure was dichotomized into daily/near-daily versus less frequent use (weekly, monthly, or less than monthly).

Daily cannabis use intensity was measured for both inhaled and oral cannabis products. Inhaled intensity was assessed as the typical number of hits per day (from a pipe, joint, bong, etc.) and oral use intensity as the number of times per day for edible, drinkable, or other oral products. Because both variables were highly skewed, hits per day were dichotomized at the median of 10 (≥10 vs. <10), and oral use per day at the median of 2 (≥2 vs. 1). Participants reporting zero hits (i.e., not inhaling cannabis), zero oral use, or “don’t know” were treated as missing for the respective variable.

Participants reported on their use of nine different cannabis forms (see Table 2). While the original response options captured frequency categories, each form was dichotomized as “yes” (any use) or “no” (did not use). Additionally, the median number of cannabis forms used was calculated for the sample.

Problematic cannabis use was assessed using the 5-item Severity of Dependence Scale (SDS) (Gossop et al., 1995), which captures psychological components of dependence such as impaired control and preoccupation with use. Total scores ranging 0–15 were converted into a binary measure at the value of 4 or higher indicating likely dependence or problematic use (Martin et al., 2006, van der Pol et al., 2013).

Three yes/no variables were tested as related to the context of cannabis use: driving under the influence of cannabis, typically using alone versus with others, and the timing of use during the day. The timing variable included options for specific times of day (e.g., morning, evening), as well as an additional option for use at any time of day or night. For our analysis, we contrasted any time use with use at specific times of day, which may reflect more intentional or structured use.

#### 2.2.3 Past 90-day health and substance use

Anxiety and depressive symptoms were assessed using the 7-item Generalized Anxiety Disorder scale (GAD-7; Spitzer et al., 2006) and the 8-item Patient Health Questionnaire scale (PHQ-8; Kroenke et al., 2009). Both scales use Likert-type response options, with total scores ranging from 0 to 21 (GAD-7) and 0–24 (PHQ-8), respectively. For both measures, scores were dichotomized at a clinical cutoff of 10 to indicate moderate-to-severe symptoms versus minimal-to-mild symptoms, consistent with prior research (Spitzer et al., 2006, Kroenke et al., 2009).

Physical health over the past 90 days was assessed with items on current pain and sleep problems. Current non-minor pain was measured with a question adapted from the Brief Pain Inventory (Short Form) (Cleeland, 2009). Sleep problems were assessed separately as a four-category variable, which was collapsed into two categories: frequent sleep problems (“in half the days” or “nearly every day”) and infrequent/none (“none” or “several days”).

The survey also asked about substance use in the past 90 days (yes/no). Alcohol use was assessed separately, however due to low prevalence of other substances, we created composite categories for tobacco products (cigarettes, cigars, e-cigarettes, hookah), and illicit drugs (LSD, mushrooms, MDMA, cocaine, crack, methamphetamine).

### 2.3 Analysis

We examined baseline differences between mothers and fathers across a range of sociodemographic and baseline characteristics to identify potential confounders for adjusted analyses. Chi-square tests were used to assess differences in categorical variables by parent’s sex; a *t*-test was used for age and age at cannabis use onset. To evaluate which baseline characteristics were independently associated with parent’s sex, we conducted a multivariable logistic regression model with parent’s sex as the outcome variable. The model included all variables that were significant in bivariate analyses (p < .05), as well as age and race. Variables that remained significantly associated with parent’s sex in the multivariable model, as well as age and race, were retained as covariates in subsequent adjusted models.

We then examined associations between parent’s sex (exposure) and characteristics related to recent cannabis use, health, and substance use, using both unadjusted and adjusted logistic regression models. Multicollinearity among covariates was assessed prior to adjusted analyses using variance inflation factors (VIF). All analyses were conducted in SPSS version 30.0, using a significance level of p < 0.05.

## 3 Results

### 3.1 Sociodemographic and baseline characteristics

Table 1 presents sociodemographic and baseline differences between mothers and fathers who lived with children and used cannabis as a medical cannabis patient. Of the 372 parents in the sample, 234 (62.9 %) were mothers and 138 (37.1 %) were fathers. Overall, participants were middle-aged (mean = 42.62 years; SD = 10.18), and the majority identified as non-Hispanic White (79.8 %). Among participants of color (20.2 %), racial/ethnic identities included non-Hispanic Black/African American (13.7 %), Hispanic/Latino (3.8 %), non-Hispanic multiracial (2.2 %), and non-Hispanic Asian (0.5 %) (data not shown in the table).

**Table 1:** Sociodemographic and baseline characteristics of mothers and fathers who use medical cannabis.

	Total (N = 372)	Mothers (n = 234)	Fathers (n = 138)	p-value (unadjusted)	p-value (adjusted)
***Social and demographic characteristics***					
Age, mean (SD)	42.62 (10.18)	42.54 (10.23)	42.75 (10.14)	.851	.653
Non-Hispanic White, % (n)	79.8 (297)	81.2 (190)	77.5 (107)	.395	.150
Married, % (n)	57.5 (214)	53.4 (125)	64.5 (89)	**.037**	**.011**
Bachelor’s degree or above, % (n)	42.7 (159)	45.7 (107)	37.7 (52)	.130	--
Past 90-day employment, % (n)	75.3 (280)	73.9 (173)	77.5 (107)	.436	--
Annual income > $50,000, % (n)	43.7 (169)	37.3 (67)	54.1 (79)	**.002**	**.002**
Veteran, % (n)	5.6 (21)	1.7 (4)	12.3 (17)	**< .001**	**< .001**
Any arrest, % (n)	32.5 (121)	20.5 (48)	52.9 (73)	**< .001**	**< .001**
Cannabis possession arrest, % (n)	11.3 (42)	6.4 (15)	19.6 (27)	**< .001**	.366
***Lifetime substance use***					
Age at cannabis use onset, mean (SD)	18.25 (8.25)	18.31 (7.51)	18.13 (9.39)	.837	--
Cannabis-naïve at medical certification, % (n)	9.1 (34)	9.0 (21)	9.4 (13)	.885	--
History of daily or regular cannabis use, % (n)	84.7 (315)	81.6 (191)	89.9 (124)	**.033**	.191
History of opioid misuse, % (n)	35.2 (131)	28.6 (67)	46.4 (64)	**< .001**	**.013**
***Lifetime health***					
Lifetime diagnosis of PTSD, % (n)	31.7 (118)	36.8 (86)	23.2 (32)	**.007**	**.003**
Lifetime diagnosis of neuropathic, chronic, or intractable pain, % (n)	53.5 (199)	54.3 (127)	52.2 (72)	.695	--
Lifetime diagnosis of drug or alcohol dependence, % (n)	15.9 (59)	12.8 (30)	21.0 (29)	**.037**	.658
Qualifying conditions for medical cannabis:					
Anxiety disorders, % (n)	75.8 (282)	79.9 (187)	68.8 (95)	**.016**	.127
Chronic pain, % (n)	50.0 (186)	50.4 (118)	49.3 (68)	.830	--
PTSD, % (n)	26.9 (100)	30.3 (71)	21.0 (29)	.050	--
Opioid use disorder, % (n)	9.4 (35)	7.7 (18)	12.3 (17)	.140	--

SD=standard deviation; PTSD=post-traumatic stress disorder

Notes:

1) Bolded p-values indicate statistical significance at p < .05.

2) Sample size for age at cannabis use onset is n = 371 due to missing data.

3) Unadjusted p-values are from bivariate analyses, including chi-square tests for categorical variables and *t*-test for age and age at cannabis use onset.

4) The final column shows p-values for adjusted odds ratios from a multivariable logistic regression model with parent’s sex as the outcome. Variables included in the model display p-values; variables not included are marked with dashes.

Age, race, education, and employment levels did not differ significantly by parent’s sex. However, bivariate analyses revealed that, compared to fathers, mothers were significantly less likely to be married (53.4 % vs. 64.5 %), have an annual income over $50,000 (37.3 % vs. 54.1 %), report veteran status (1.7 % vs. 12.3 %), and have a history of arrest (20.5 % vs. 52.9 %), including cannabis possession arrest (6.4 % vs. 19.6 %).

With respect to lifetime substance use and health, bivariate analyses showed no differences in the mean age at cannabis use onset or cannabis-naïve status by parent’s sex (Table 1). However, mothers were significantly less likely to report a history of daily or regular cannabis use (81.6 % vs. 89.9 %), lifetime opioid misuse (28.6 % vs. 46.4 %), or lifetime diagnosis of drug or alcohol dependence (12.8 % vs. 21.0 %). In contrast, mothers had a significantly higher prevalence of lifetime PTSD diagnosis (36.8 % vs. 23.2 %) and were significantly more likely to report anxiety disorders as a qualifying condition for medical cannabis (79.9 % vs. 68.8 %).

In a multivariable model that included all sociodemographic and baseline characteristics significant at the bivariate level, along with age and race, six characteristics—marital status, higher income, veteran status, history of arrest, PTSD diagnosis, and lifetime opioid misuse—remained independently associated with parent’s sex (Table 1). These variables, along with age and race, were included as covariates in subsequent multivariable models examining associations between parent’s sex and recent health and cannabis use characteristics. Collinearity diagnostics for the adjusted models indicated no significant multicollinearity among covariates and parent’s sex, with variance inflation factor (VIF) values ranging from 1.07 to 1.34.

### 3.2 Past 90-day cannabis use

In the past 90 days, 84.7 % of parents used cannabis daily or near-daily, and 15.3 % used less frequently (12.9 % weekly, 2.2 % monthly, and 0.3 % less than monthly). Daily/near-daily use did not significantly differ between mothers (84.6 %) and fathers (84.8 %) (Table 2). Parent’s sex was also not associated with higher use intensity for inhaled use (≥10 daily hits) or oral use (≥2 times per day) (Table 2). Overall, fewer than one in five parents (16.9 %) met the threshold for problematic cannabis use as measured by the Severity of Dependence Scale, with no statistically significant difference between mothers (15.0 %) and fathers (20.3 %).

**Table 2:** Past 90-day cannabis use among mothers and fathers who use medical cannabis.

	Total sample (N = 372)% (n)	Mothers (n = 234)% (n)	Fathers (n = 138)% (n)	Mothers vs. Fathers	
				UOR [95 % CI]	AOR [95 % CI]
***Cannabis frequency and problematic use***					
Daily/near-daily cannabis use	84.7 (315)	84.6 (198)	84.8 (117)	0.99 [0.55, 1.77]	1.04 [0.52, 2.10]
≥ 10 hits per day (n = 315)	53.0 (167)	51.8 (101)	55.0 (66)	0.88 [0.56, 1.39]	0.91 [0.51, 1.64]
≥ 2 oral uses per day (n = 253)	58.9 (149)	55.1 (87)	65.3 (62)	0.65 [0.39, 1.10]	0.78 [0.41, 1.48]
Problematic cannabis use (SDS≥4)	16.9 (63)	15.0 (35)	20.3 (28)	0.69 [0.40, 1.20]	0.67 [0.35, 1.26]
***Cannabis forms***					
Flower/buds	80.1 (298)	76.1 (178)	87.0 (120)	0.48 [0.27, 0.85]*	0.57 [0.29, 1.11]
CO_2_ vape oil	73.9 (275)	73.9 (173)	73.9 (102)	1.00 [0.62, 1.62]	0.94 [0.53, 1.68]
Concentrates	38.2 (142)	33.3 (78)	46.4 (64)	0.58 [0.38, 0.89]*	0.56 [0.33, 0.96]*
Moon rocks/caviar	19.1 (71)	16.2 (38)	23.9 (33)	0.62 [0.37, 1.04]	0.71 [0.37, 1.37]
Liquid sugar	59.7 (222)	56.0 (131)	65.9 (91)	0.66 [0.43, 1.02]	0.66 [0.39, 1.12]
Rick Simpson Oil	36.8 (137)	34.6 (81)	40.6 (56)	0.76 [0.50, 1.20]	0.79 [0.47, 1.32]
Capsules	34.1 (127)	37.6 (88)	28.3 (39)	1.53 [0.97, 2.41]	1.87 [1.09, 3.19]*
Tinctures	37.6 (140)	41.9 (98)	30.4 (42)	1.65 [1.05, 2.57]*	1.71 [1.01, 2.87]*
Creams/Topicals	35.8 (133)	39.3 (92)	29.7 (41)	1.53 [0.98, 2.40]	1.58 [0.94, 2.67]
***Context of cannabis use***					
Using alone (n = 371)	75.5 (280)	78.5 (183)	70.3 (97)	1.55 [0.96, 2.50]	1.79 [1.02, 3.14]*
Using at specific times of day	53.2 (198)	58.5 (137)	44.2 (61)	1.78 [1.17, 2.73]**	1.85 [1.08, 3.16]*
Driving under the influence (n = 347)	41.8 (145)	39.3 (86)	46.1 (59)	0.76 [0.49, 1.18]	0.85 [0.50, 1.46]

*p < 0.05; **p < 0.01

UOR=unadjusted odds ratio; AOR=adjusted odds ratio; CI=confidence interval; SDS=Severity of Dependence Scale

Notes:

1) Sample sizes for four variables (hits, oral uses, using alone, and driving under the influence) are smaller than the total N = 372 due to missing data and/or ineligibility.

2) Adjusted models controlled for age, race, marital status, income, veteran status, history of arrest, history of opioid misuse, and lifetime PTSD.

However, significant sex differences emerged in the use of cannabis forms. While the median number of cannabis forms used in the past 90 days was similar for mothers and fathers (4 of 9; data not shown in the table), mothers had lower odds of using concentrates (AOR = 0.56, 95 % CI: 0.33–0.96) and higher odds of using oral forms such as capsules (AOR = 1.87, 95 % CI: 1.09–3.19) and tinctures (AOR = 1.71, 95 % CI: 1.01–2.87). In unadjusted models, mothers also had lower odds of using cannabis flower, though this association was not significant after adjustment.

Differences also emerged in the context of cannabis use. Adjusted models indicated that, compared to fathers, mothers had higher odds of using cannabis alone versus with others (AOR = 1.79, 95 % CI: 1.02–3.14) and of using cannabis at specific times of day versus any time (AOR = 1.85, 95 % CI: 1.08–3.16). However, driving under the influence of cannabis in the past 90 days did not differ by parent’s sex (Table 2).

### 3.3 Past 90-day health and substance use

Compared to fathers, mothers had significantly higher odds of frequent sleep problems in the past 90 days (AOR = 1.96, 95 % CI: 1.18–3.25) (Table 3). Mothers also reported significantly higher levels of moderate-to-severe anxiety (AOR = 1.91, 95 % CI: 1.14–3.20). Although mothers showed higher levels of depression than fathers in unadjusted models, these differences were not statistically significant after adjustment.

**Table 3:** Past 90-day health and substance use among mothers and fathers who use medical cannabis.

	Total sample (N = 372)% (n)	Mothers (n = 234)% (n)	Fathers (n = 138)% (n)	Mothers vs. Fathers	
				UOR [95 %CI]	AOR [95 %CI]
***Health symptoms***					
Frequent sleep problems	43.8 (163)	49.6 (116)	34.1 (47)	1.90 [1.23, 2.94]**	1.96 [1.18, 3.25]*
Current non-minor pain	54.6 (203)	56.8 (133)	50.7 (70)	1.28 [0.84, 1.95]	1.00 [0.60, 1.66]
Moderate-to-severe anxiety (GAD−7 ≥10)	46.0 (171)	50.9 (119)	37.7 (52)	1.71 [1.11, 2.63]*	1.91 [1.14, 3.20]*
Moderate-to-severe depression (PHQ−8 ≥10)	35.5 (132)	39.3 (92)	29.0 (40)	1.59 [1.01, 2.49]*	1.28 [0.75, 2.17]
***Substance use***					
Alcohol	32.3 (120)	27.8 (65)	39.9 (55)	0.58 [0.37, 0.91]*	0.64 [0.38, 1.08]
Tobacco products	26.9 (100)	23.9 (56)	31.9 (44)	0.67 [0.42, 1.07]	0.60 [0.34, 1.08]
Illicit drugs	4.8 (18)	2.6 (6)	8.7 (12)	0.28 [0.10, 0.75]*	0.25 [0.08, 0.82]*

*p < 0.01; **p < 0.01

UOR=unadjusted odds ratio, AOR=adjusted odds ratio; CI=confidence interval; GAD-7 = 7-item Generalized Anxiety Disorder Scale; PHQ-8 = 8-item Patient Health Questionnaire.

Note: Adjusted models controlled for age, race, marital status, income, veteran status, history of arrest, history of opioid misuse, and lifetime PTSD.

Finally, the sample reported relatively low levels of past 90-day illicit drug use (4.8 %), with mothers having significantly lower odds of illicit drug use compared to fathers (AOR = 0.25, 95 % CI: 0.08–0.82). No significant differences were observed between mothers and fathers in tobacco product use. Although mothers reported lower rates of alcohol use in unadjusted models, this association was not significant after adjustment (Table 3).

## 4 Discussion

This study identified notable gender differences among medical cannabis patients who are actively parenting. Our primary finding indicates that, despite less severe histories of substance use and lower exposure to law enforcement compared to fathers, mothers faced greater socioeconomic vulnerability, a higher prevalence of anxiety and sleep problems, and more discreet patterns of cannabis use. Conversely, fathers reported more extensive lifetime adversity, but fewer anxiety symptoms and more flexible cannabis use. To our knowledge, this is the first study to examine these differences specifically among parents who use medical cannabis. While some patterns may reflect broader gender dynamics, they remain important in a parenting context because of potential effects on caregiving. Additionally, even when parental use patterns resemble those of nonparent medical cannabis patients, the presence of children adds distinct implications for family functioning and child well-being.

Mothers in our sample reported elevated anxiety and disrupted sleep, as well as a higher prevalence of PTSD diagnosis compared to fathers. These patterns may reflect both general sex differences in mental health observed in U.S. populations (McLean et al., 2011, Olff, 2017) and possible challenges associated with caregiving roles. While our data cannot establish the sources of these differences, findings align with broader literature on gender disparities in mental health among U.S. parents, where mothers experience higher prevalence of any mental disorder (Stambaugh et al., 2017) and greater parental stress and fatigue (Musick et al., 2016) than fathers.

Our findings also suggest that these differences may intersect with structural disadvantages. Compared to fathers, a greater proportion of mothers in our sample were unmarried (likely single parents) and had lower incomes. These constraints could shape how mothers use cannabis. While their use patterns—including higher use of oral forms, such as tinctures and capsules, lower use of concentrates, and using alone or at specific times—may appear responsible or therapeutic, they might also reflect practical considerations (e.g., limited time) or efforts to avoid stigma. Social expectations around motherhood and substance use expose mothers to heightened stigma and surveillance (Greene et al., 2023, Withanarachchie et al., 2025), which may in turn contribute to distress and more concealed patterns of cannabis use.

Fathers, by contrast, more frequently reported past opioid misuse, criminal justice involvement, and veteran status—characteristics often associated with marginalization or trauma. Yet, they had a lower prevalence of PTSD and anxiety and comparable levels of depression. Moreover, fathers exhibited more flexible cannabis use patterns, being more likely to consume concentrated cannabis forms and use cannabis at any time of the day. This aligns with research showing that men tend to use more potent and less discreet cannabis products compared to women (Cuttler et al., 2016). While such behaviors might suggest higher-risk use, they may also reflect stabilization or harm reduction following prior opioid misuse. This interpretation aligns with research showing that some medical cannabis patients use cannabis as a substitute for more harmful substances (Fedorova et al., 2020, Lucas et al., 2019, Valdez et al., 2025). Future research should explore whether better mental health and more flexible use patterns among fathers are related to different experiences of stigma compared to mothers, fathers’ access to buffering resources, such as marital support, or a secondary caregiving role.

Taken together, these findings underscore the gendered nature of cannabis use within parenting contexts. While fathers may be transitioning from more harmful substance use, including opioid misuse, and leveraging cannabis as a stabilizing tool, mothers are likely navigating cannabis use within contexts of structural vulnerability and intense caregiving demands. The contrasting profiles suggest that cannabis may serve different roles across genders, affected by caregiving structure, stigma, and access to resources. Furthermore, these dynamics may carry certain implications for children. While discreet maternal use may reduce children’s direct exposure, it may increase maternal stress due to secrecy, with potential negative effects on children. Conversely, more visible paternal use may contribute to cannabis use behavior modeling.

Importantly, less than one-fifth of parents in our study (17 %) reported problematic cannabis use, with no significant differences between mothers and fathers. This is lower than the prevalence of cannabis use disorder among a broader group of people who use medicinal cannabis, estimated at 25 % (CI: 18–33 %) by a recent systematic review and meta-analysis (Dawson et al., 2024). The similar rates of problematic use among mothers and fathers, despite different patterns of use, suggest distinct modes of integrating cannabis into daily life, rather than differences in misuse or risk.

These findings have important implications for how parental cannabis use is understood and addressed in public health and policy. Our study demonstrated that mothers and fathers use cannabis in distinct ways and future research is warranted to elucidate how gendered parental use is shaped by caregiving roles, stigma, and access to resources. Moreover, our study suggests that gender-responsive support programs could better address the different needs of mothers and fathers. Specifically, mothers may benefit from interventions focused on mental health and expanded social support, particularly to mitigate stigma’s effects. In contrast, fathers may benefit from increased awareness of safer cannabis use practices, including guidance on product choice and discretion. Additionally, while most parents in our study showed no signs of dependence, suggesting that cannabis may serve a therapeutic or stabilizing role, further research should explore the subset of parents with problematic use to understand contributing factors and inform effective interventions. Longitudinal and qualitative studies are especially well-suited to explore how cannabis use evolves alongside caregiving, mental health, and family relationships. Furthermore, public health messaging, clinical care, and social services should consider parenting realities within medical cannabis use. Finally, although we focused on parents to reduce heterogeneity and highlight caregiving-relevant contexts, future research could compare parents and nonparents who use medical cannabis to clarify which patterns are specific to parenting.

Several limitations of the study should be noted. All data were self-reported, which introduces potential recall bias. We do not have data on children’s ages or parenting arrangements (e.g., shared custody, cohabitation) or co-parent cannabis use, potentially obscuring differences in family support. Furthermore, the cross-sectional nature of our analysis limits our ability to determine temporal relationships between variables. Additionally, parent’s sex was measured as a binary female/male variable to distinguish between mothers and fathers; however, this approach does not capture gender diversity or the complexity of caregiving roles beyond a binary framework. Since our survey did not ask if other members of their household were enrolled in the study, we also cannot rule out that more than one parent from the same family participated (i.e., parent dyads), which may introduce non-independence into the sample. Moreover, our sample was not randomly selected and may not be fully generalizable; however, the distribution of key qualifying health conditions, including anxiety disorders, chronic pain, and PTSD, closely mirrors that of Pennsylvania’s broader population of medical cannabis patients (Commonwealth of Pennsylvania, 2025). Finally, because opioid use disorder is a qualifying condition for medical cannabis in Pennsylvania, our sample may differ from the general U.S. population of medical cannabis patients, with higher prevalence of lifetime opioid misuse and, correspondingly, higher likelihood of criminal justice involvement (Winkelman et al., 2018). These characteristics should be considered when interpreting our findings.

In conclusion, our study found that mothers who use medical cannabis faced greater socioeconomic vulnerability, elevated anxiety and sleep disturbances, and used cannabis more discreetly, while fathers reported more flexible use patterns and fewer anxiety symptoms despite greater lifetime adversity. For mothers, cannabis may represent a coping strategy shaped by economic hardship, trauma, and a need for discretion; for fathers, it may serve as a harm reduction or substitution strategy. These gendered patterns highlight the need to understand parental cannabis use within the structural and social realities of caregiving. Public health and clinical responses should reflect these differences, and future research should further explore how caregiving roles, stigma, and systemic constraints shape cannabis use among parents.

## Author disclosures

The authors report funding by Agronomed Biologics (owned by Verano).

## CRediT authorship contribution statement

**Janna Ataiants:** Writing – review & editing, Writing – original draft, Methodology, Investigation, Formal analysis, Conceptualization. **Ekaterina V. Fedorova:** Writing – review & editing. **Ojaswini D. Parab:** Writing – review & editing, Project administration. **Maddy Finkelstein:** Writing – review & editing. **Elizabeth S. Valdez:** Writing – review & editing, Conceptualization. **Olivia Cordingley:** Writing – review & editing. **Stephen E. Lankenau:** Writing – review & editing, Conceptualization.

## Role of funding source

This work was supported by a multi-year research agreement between Drexel University and Agronomed Biologics, which is owned by Verano (a multi-state cannabis company).

## Declaration of Competing Interest

The authors report that this research was funded by Agronomed Biologics, which is owned by Verano (a multi-state cannabis company). The funder had no role in the study design, the analysis or interpretation of the data, the preparation of this manuscript, or the decision to submit the manuscript for publication.
